# Research on the Microstructure and Mechanical Properties of Automatically Welded Martensitic Stainless Steel Joints for Thick Plates

**DOI:** 10.3390/ma19122507

**Published:** 2026-06-10

**Authors:** Yunxia Chen, Yunwang Ding, Shanshan Lyu, Zesong Chen

**Affiliations:** 1 School of Intelligent Manufacturing and Control Engineering, Shanghai Polytechnic University, Shanghai 201209, China; 20251513193@sspu.edu.cn (Y.D.);; 2 School of Materials Science and Engineering, Shanghai Jiao Tong University, Shanghai 200240, China

**Keywords:** 20Cr11W2VTaSi martensitic heat-resistant steel, weld microstructure, narrow-gap TIG welding, high-temperature tensile strength, impact toughness

## Abstract

To address the performance degradation associated with retained high-temperature δ-ferrite in welded joints of high-silicon 20Cr11W2VTaSi steel—a candidate structural material for spallation targets in Accelerator Driven Subcritical Systems—this study systematically investigates the microstructural evolution and mechanical behavior of 20 mm-thick forged joints produced via automated tungsten inert gas (TIG) welding using a 7° U-groove narrow-gap configuration. Results demonstrate that the narrow-gap process—featuring reduced filler metal deposition and low heat input—is believed to suppress macrosegregation of ferrite-stabilizing elements (e.g., Cr, Si, Mo). As a result, the δ-ferrite content in the weld metal is constrained, exhibiting a fine, dispersed, worm-like morphology embedded within a uniform matrix of tempered martensite. Microhardness mapping confirms homogeneous hardness distribution across the joint, closely matching that of the base metal, with no statistically significant localized softening zones identified. Mechanical characterization reveals an optimal balance of strength and toughness: the joint achieves a room-temperature tensile strength of 820 MPa and retains 436 MPa at 550 °C; moreover, the Charpy impact energy at the weld center reaches 171.2 J.

## 1. Introduction

The development of advanced nuclear energy systems—particularly Accelerator Driven Subcritical Systems (ADSs)—represents a strategic pathway toward enhancing global energy sustainability and enabling efficient transmutation of long-lived nuclear waste [1,2,3]. As the central component of the ADS, the spallation target operates under exceptionally demanding conditions, including elevated temperatures (>500 °C), high-intensity neutron irradiation (up to ~10 dpa/year), and prolonged exposure to corrosive liquid lead–bismuth eutectic (LBE) coolant. Under such extreme environments, the structural integrity and operational reliability of target assemblies are critically dependent on the thermo-mechanical stability and irradiation-resistant performance of constituent materials [4]. 20Cr11W2VTaSi steel (commonly designated SIMP steel), a newly developed high-silicon martensitic heat-resistant alloy, has been identified as a leading candidate for spallation target fabrication. Its tailored composition—especially the elevated silicon content (≈2.0 wt.%)—confers superior high-temperature oxidation resistance, enhanced thermal conductivity, and markedly improved chemical compatibility with LBE relative to conventional ferritic/martensitic steels such as T91 and EUROFER97 [5,6].

However, the engineering deployment of SIMP steel remains hindered by persistent metallurgical challenges associated with welding fabrication—particularly for thick-section components mandated by structural design requirements. The root cause resides in non-equilibrium solidification behavior, which governs phase evolution during rapid thermal cycling. Due to its elevated concentrations of ferrite-stabilizing elements—especially silicon, along with chromium and tungsten—the austenite phase field in SIMP steel is markedly narrowed [7]. Consequently, during weld thermal cycles, the weld metal (WM) exhibits a strong tendency to retain high-temperature δ-ferrite down to room temperature. In conventional fusion welding processes—including Shielded Metal Arc Welding (SMAW) and standard Gas Tungsten Arc Welding (GTAW) with U-groove preparations—the combination of high heat input and sluggish cooling kinetics promotes solute segregation and diffusion-controlled partitioning of ferrite stabilizers. Under these thermodynamic conditions, coarse, blocky δ-ferrite preferentially nucleates and grows at martensitic lath boundaries [8,9]. Extensive experimental evidence confirms that this retained δ-ferrite constitutes a critical microstructural liability: it induces localized hardness reduction and, more detrimentally, acts as a nucleation sink for brittle intermetallic phases—particularly the Laves phase—during high-temperature service or post-weld thermal aging. Such microstructural degradation directly compromises the Charpy impact toughness of the welded joint, thereby introducing a systemic vulnerability that undermines the structural safety and operational integrity of the ADS spallation target.

In the industrial fabrication of ADS spallation targets, the welding of thick-section SIMP steel components demands highly reliable and efficient processes. Conventional multi-pass welding techniques often suffer from low productivity, high heat input, and high defect rates, which fail to meet the stringent structural integrity requirements of the nuclear industry. The main practical and industrial contribution of this investigation is the implementation of an automated narrow-gap TIG (NG-TIG) strategy. This approach not only significantly improves deposition efficiency and reduces material consumption for 20 mm-thick plates, but also intrinsically mitigates the detrimental metallurgical degradations—specifically the retention of coarse δ-ferrite—thereby directly fulfilling the industrial need for robust, long-life spallation target assemblies.

To mitigate these metallurgical challenges, precise control of heat input and interpass cooling rate during welding is essential to govern both the volume fraction and morphological evolution of retained δ-ferrite. Although post-weld heat treatment (PWHT) effectively alleviates residual stresses, it exhibits negligible efficacy in dissolving coarse, as-solidified δ-ferrite due to its thermodynamic stability and sluggish diffusion kinetics below the δ → γ transformation temperature [10]. Consequently, process-level innovation—rather than post-fabrication correction—is indispensable for microstructural control. Narrow-gap automatic Tungsten Inert Gas (NG-TIG) welding presents a technically viable solution: its geometric constraint inherently limits filler metal deposition and total thermal energy input per pass. Critically, the enhanced heat extraction at the sidewalls—termed the “sidewall cooling effect”—imposes rapid solidification conditions that kinetically suppress long-range solute diffusion and impede the coarsening of blocky δ-ferrite [11,12]. While NG-TIG has been successfully implemented for thick-section welds in conventional power plant steels (e.g., P92 and NF709), its application to 20 mm-thick SIMP steel remains underexplored. In particular, the quantitative correlations among narrow-gap cooling kinetics, the topological characteristics (e.g., aspect ratio, spatial distribution, and interconnectivity) of retained δ-ferrite, and the resultant Charpy V-notch fracture toughness have yet to be established experimentally. Furthermore, advanced non-destructive testing (NDT) approaches are becoming essential for quantifying microstructural heterogeneity in high-strength steels. For instance, recent studies have successfully employed pulsed laser spot thermography to non-destructively monitor phase transformations (e.g., bainite/martensite variations) and estimate ultimate tensile strength in steel alloys [13]. Such quantitative inspection methods represent a critical future direction for evaluating thick-section SIMP steel components.

To address this critical knowledge gap, this study systematically investigates the microstructural evolution and mechanical behavior of 20 mm-thick SIMP steel joints. The core novelty of this work lies in the successful implementation of a newly developed matching-composition commercial filler wire, combined with an automated 7° U-groove narrow-gap TIG configuration. Moving beyond standard feasibility assessments, this research aims to demonstrate how pairing this specific novel consumable with strictly controlled low heat input is believed to suppress macrosegregation and constrain retained δ-ferrite. By establishing this specific process–material synergy, this study provides actionable, industrially viable process guidelines for the reliable manufacturing of ADS spallation target components.

## 2. Materials and Experimental Methods

### 2.1. Materials

The base material used in this study was a 20 mm-thick forged plate of 20Cr11W2VTaSi steel (SIMP steel), a high-silicon martensitic heat-resistant alloy specifically developed for structural components in ADSs. A matching-composition filler wire (diameter: 1.2 mm) was selected to ensure metallurgical continuity. The chemical composition ranges of both the base metal (BM) and filler wire are provided in Table 1. The as-received room-temperature tensile properties and Charpy V-notch impact energy of the SIMP steel BM, measured experimentally prior to welding, are summarized in Table 2.

**Figure 1:**
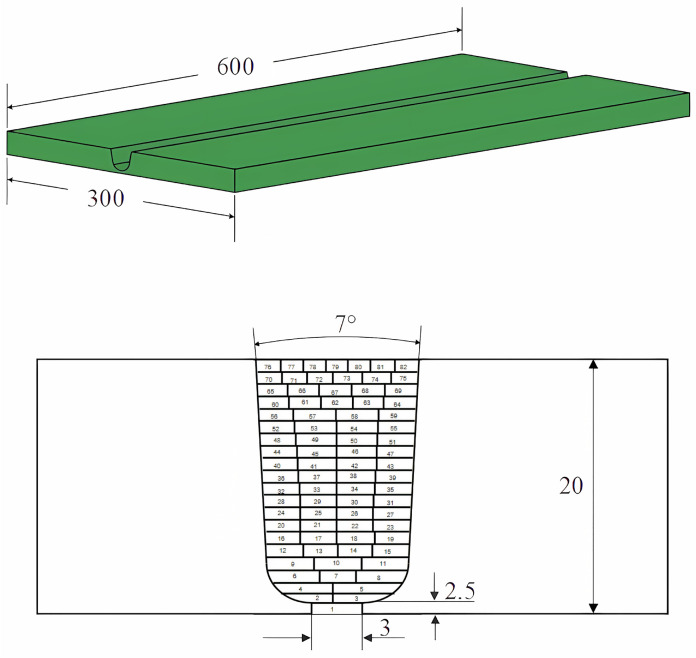
Schematic of U-groove joint assembly, weld layer arrangement, and welding sequence.

The welding procedure was executed using an automated TIG system, consisting of an EWM Tetrix 351 AC/DC (EWM GmbH, Mündersbach, Deutschland) inverter power source coupled with an ABB IRB 6700 robotic manipulator to ensure repeatable control of weld bead placement, travel speed, and thermal input. High-purity argon (99.99% Ar) served as the primary shielding gas. To constrain heat accumulation and kinetically suppress coarse δ-ferrite formation, a narrow-gap U-groove joint design was implemented—with a precise bevel angle of 7°, root face of 3 mm, and root gap of 1 mm—as illustrated in Figure 1. The full-thickness weld was deposited in 21 layers comprising 82 individual passes, using the process parameters detailed in Table 3. The process parameters detailed in Table 3 were established through a series of preliminary experimental trial welds on the SIMP steel. The methodology for parameter selection was specifically designed to satisfy two competing criteria: (1) ensuring complete sidewall fusion within the highly constrained 7° narrow-gap U-groove, and (2) strictly minimizing the overall thermal input to kinetically suppress the excessive growth of δ-ferrite. The final selected values represent the optimized operational window that achieves this thermo-mechanical balance without inducing macroscopic welding defects.

Following welding, the joints were subjected to a two-stage post-weld heat treatment (PWHT) to relieve residual stresses and homogenize the tempered martensitic microstructure in a standard electrical resistance furnace under ambient atmosphere. The thermal cycle comprised: (i) a preliminary soak at 600 °C for 20 min to ensure through-thickness thermal uniformity, followed at 1050 °C for 40 min with heating at 200 °C/h; and (ii) tempering at 760 °C for 90 min after reheating at 200 °C/h, with final air cooling to ambient temperature.

### 2.2. Nondestructive Testing and Microstructural Characterization

Prior to sectioning, the structural integrity of all welded joints was rigorously assessed via visual inspection and radiographic testing (RT), conducted in strict compliance with ASME Boiler and Pressure Vessel Code Section V (2004 Edition). Joints exhibiting no surface-breaking indications or internal volumetric defects—such as porosity, slag inclusions, or lack of fusion—were retained for subsequent metallurgical and mechanical evaluation.

Metallographic specimens were extracted from the mid-thickness region of the stable weld zone to ensure representative sampling of the fusion boundary and Heat-Affected Zone (HAZ) microstructures. Specimens were sequentially ground using SiC abrasive papers (up to 2500 grit) and polished with diamond suspension (3 µm followed by 1 µm), followed by final colloidal silica polishing to eliminate mechanical deformation artifacts. Microstructural characterization was performed using a Zeiss Imager A2 optical microscope (OM) (Carl Zeiss AG, Oberkochen, Germany) and a JEOL JSM-7600F field-emission scanning electron microscope (FE-SEM, JEOL Ltd. Tokyo, Japan), equipped with energy-dispersive X-ray spectroscopy (EDS). All sample preparation and imaging procedures strictly adhered to ASTM E3 [14] (Standard Guide for Preparation of Metallographic Specimens). Image analysis software (ImageJ v1.54f) was employed to quantitatively determine the volume fraction, aspect ratio, and spatial distribution of retained δ-ferrite. The quantitative analysis was performed on 5 randomly selected optical fields of view at 500× magnification. Standard grayscale thresholding was applied to segment the unetched bright δ-ferrite from the darker martensite matrix.

### 2.3. Mechanical Property Evaluation

Mechanical properties of the joints were evaluated using standardized specimens. Tensile tests perpendicular to the welding direction were conducted at room temperature per AWS B4.0M [15] and at elevated temperatures (250, 300, 350, 400, 450, 500, and 550 °C) per ASTM E21 [16] on a Zwick Roell Z100 universal testing machine, using three replicates for each condition. Room-temperature Charpy V-notch impact tests (four specimens per group) were performed following AWS B4.0M, with the notch centerlines located at the weld center and 2 mm from the fusion line for the weld metal and HAZ, respectively. Side-bend tests (four specimens) perpendicular to the welding direction were carried out according to AWS B4.0M. Vickers microhardness profiles across the full cross-section were measured at room temperature using an automatic hardness tester compliant with AWS B4.0M.

## 3. Results and Discussion

### 3.1. Phase Transformation Behavior and Microstructure

**Figure 2:**
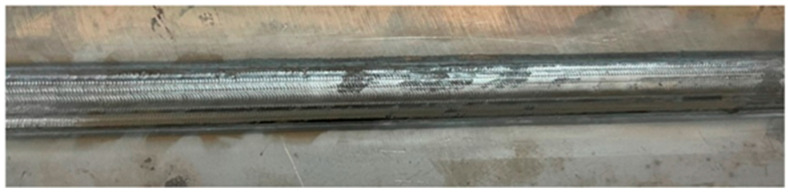
Surface appearance of the narrow-gap TIG welded joint.

The macrostructural integrity and microstructural evolution of the welded joint are predominantly governed by the groove geometry and the associated thermal input profile. Figure 2 presents the macrostructure of the full-penetration weld joint fabricated via automated 7° narrow-gap U-groove TIG welding. As observed, the joint exhibits complete sidewall fusion, uniform bead contour, and absence of process-induced defects—including lack of fusion, porosity, cracks, or excessive spatter—demonstrating robust process stability and geometric reproducibility.

**Figure 3:**
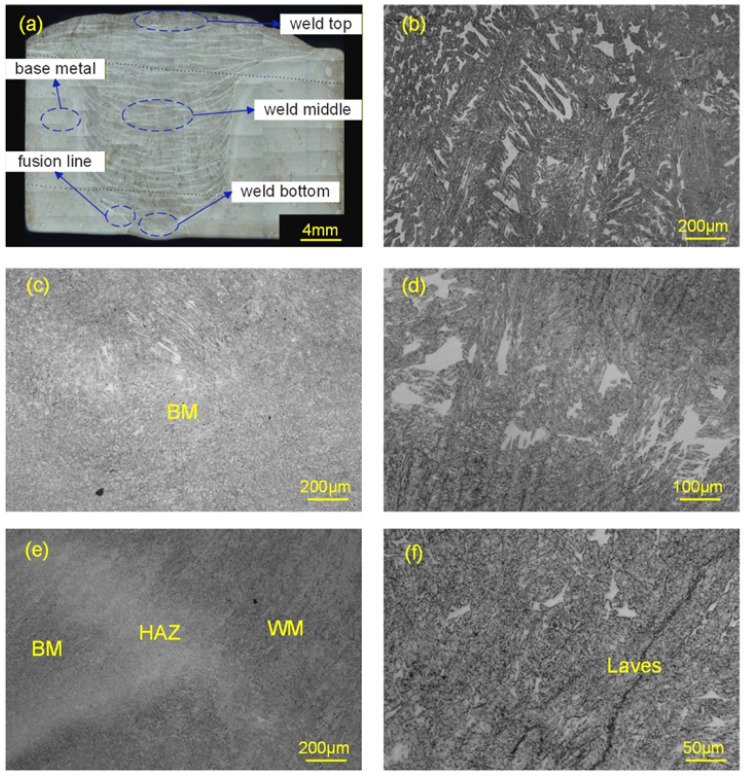
Microstructures of the automatic welded joint: (**a**) Macrograph of the welded joint showing sampling locations; (**b**) Weld top region; (**c**) BM region; (**d**) Weld middle region; (**e**) Fusion line region; (**f**) Weld bottom region. (BM: Base Metal; HAZ: Heat-Affected Zone; WM: Weld Metal).

Figure 3 presents the representative microstructures across the joint. The SIMP steel BM displays a homogeneous tempered martensitic microstructure, consisting of lath martensite packets tempered with fine M23C6 and MX-type carbides/nitrides [17]. In the WM, the elevated silicon content (1.8–2.1 wt.%, a potent ferrite stabilizer) substantially suppresses the austenite phase field in the Fe–Cr–Si–W–V–Ta system, thereby promoting the retention of high-temperature δ-ferrite during non-equilibrium solidification. Nevertheless, the narrow-gap automated TIG welding process employed—characterized by minimal filler deposition per pass and strictly controlled low thermal input—established a steep thermal gradient at the fusion boundary, enabling rapid interfacial cooling and kinetically suppressing excessive δ-ferrite growth.

This rapid interfacial cooling profoundly influenced the non-equilibrium solidification pathway. First, the substantial undercooling (ΔT ≈ 350 °C) drastically reduced the high-temperature residence time at the solid–liquid interface, thereby suppressing long-range diffusion and macrosegregation of ferrite-stabilizing elements—particularly Si and Cr—thus diminishing the thermodynamic driving force for δ-ferrite growth [18]. Second, the elevated undercooling markedly increased the nucleation density of δ-ferrite, favoring its precipitation as fine, discontinuous, worm-like or island-shaped particles preferentially at prior austenite grain boundaries and lath packet boundaries [19]. Quantitative image analysis revealed a controlled δ-ferrite volume fraction of 19.7 ± 1.2% in the WM, with the matrix comprising uniformly distributed, fine tempered martensite laths—effectively precluding the formation of coarse, continuous blocky δ-ferrite networks commonly observed in conventional high-heat-input welding processes.

### 3.2. Strengthening Mechanism and Microhardness Distribution

**Figure 4:**
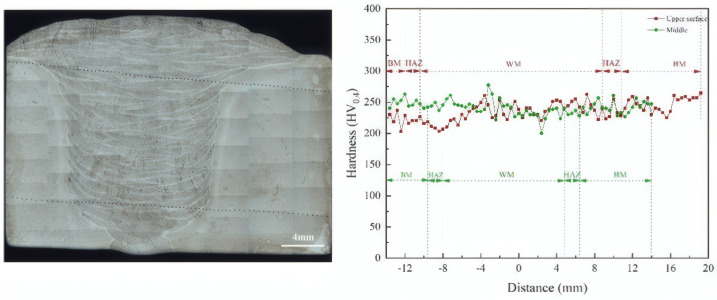
Microhardness distribution across the welded joint cross-section.

Welded joints of high-silicon martensitic heat-resistant steel often suffer from local softening due to the aggregation of δ-ferrite, which has become a bottleneck restricting their mechanical properties. Figure 4 shows the Vickers hardness distribution curve along the thickness of the joint. The test results indicate that the narrow-gap automatic welded joint exhibits a high degree of mechanical uniformity. The average hardness of the weld zone was maintained at approximately 236 HV, matching well with the hardness of the BM and the HAZ. The curve fluctuates smoothly without any distinct softening regions.

**Figure 5:**
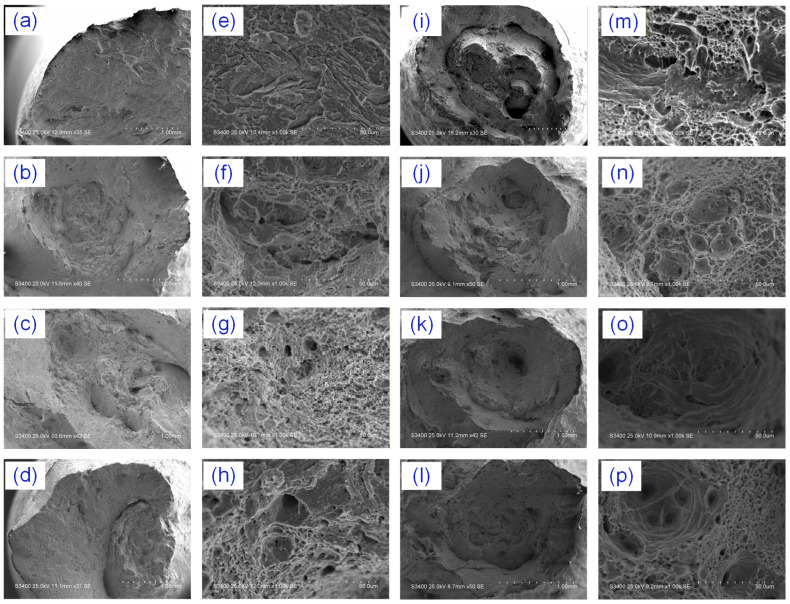
SEM fractographs of tensile specimens tested at different temperatures: (**a**,**e**) Room temperature; (**b**,**f**) 250 °C; (**c**,**g**) 300 °C; (**d**,**h**) 350 °C; (**i**,**m**) 400 °C; (**j**,**n**) 450 °C; (**k**,**o**) 500 °C; (**l**,**p**) 550 °C.

The microstructural and microhardness homogeneity directly underpins the mechanical performance shown in Figure 5: the joint achieved a room-temperature tensile strength of 820 MPa and retained 436 MPa at 550 °C, representing 53.2% of its ambient-temperature strength, and retained 436 MPa at 550 °C, representing 53.2% of its ambient-temperature strength. This exceptional strength retention demonstrates the joint’s robust load-bearing capacity and microstructural stability at elevated temperatures. This exceptional strength retention is governed by two synergistic strengthening mechanisms. First, the rapid interfacial cooling inherent to the narrow-gap automated TIG process refined both the prior austenite grain size (from ~85 μm in BM to ~22 μm in WM) and the martensite lath packet dimensions, thereby increasing the density of high-angle grain boundaries and lath interfaces. These interfaces act as potent barriers to dislocation motion, elevating the yield strength via the Hall–Petch relationship [20,21,22]. Second, a hierarchical microstructural constraint emerged from the spatial arrangement of the dual-phase constituents: the fine, discontinuous δ-ferrite particles, uniformly dispersed within the tempered martensite matrix, generated localized geometric constraints that impeded preferential plastic flow in the softer ferrite phase. This enforced deformation compatibility across micro-regions, enabling uniform load sharing and preventing premature strain localization, thereby sustaining tensile strength equivalent to the BM.

### 3.3. Tensile Properties and Fracture Behavior

To assess the joint’s load-bearing capacity across its operational temperature envelope—spanning ambient conditions to the maximum design temperature of 550 °C for ADS spallation targets—tensile tests were performed at 20 °C and 550 °C, respectively. The mechanical properties, including yield strength (Rp0.2), ultimate tensile strength (Rm), and elongation to failure, are summarized in Table 4.

Integrated with stress–strain curve analysis, the joint exhibited stable mechanical response across both strength and ductility metrics. At 20 °C, specimens achieved a tensile strength of 820 MPa and a 0.2% offset yield strength (Rp0.2) of 616 MPa, with uniform elongation (Agu) of 12.4% and total elongation to fracture (A5) of 23.7%. This high ductility—exceeding the minimum requirement of 20% specified in ASME BPVC Section II Part D for martensitic steels—confirms robust plastic deformation capacity and low susceptibility to catastrophic brittle fracture under ambient service loads. Upon heating to 550 °C, the material displayed thermally activated softening consistent with dislocation mobility enhancement; nevertheless, the joint retained a tensile strength of 436 MPa (53.2% retention) and Rp0.2 of 368 MPa (60.1% retention), satisfying the ASME BPVC Section II Part D criterion of ≥50% strength retention at design temperature. These results demonstrate that the narrow-gap automated TIG welded joint meets the structural integrity requirements for ADS spallation target applications under combined thermal–mechanical loading.

Regarding fracture behavior, macroscopic examination confirmed that all tensile specimens failed exclusively within the WM—specifically at the fusion boundary region—demonstrating full joint utilization without premature failure in the BM or HAZ. This fracture localization indicates that the weld zone, while representing the mechanically weakest link (as evidenced by its marginally lower Rp0.2 and Rm values relative to BM), achieves a joint efficiency of 99.5% for ultimate tensile strength (Rm_Weld/Rm_BM = 436/438 ≈ 0.995 at 550 °C; 820/849 ≈ 0.966 at 20 °C), confirming near-parity with the BM. This exceptional efficiency arises from the synergistic microstructural optimization detailed previously: (i) rapid solidification-induced grain refinement elevated dislocation slip resistance via increased interfacial area density; and (ii) the fine, discontinuous δ-ferrite particles, uniformly embedded in the tempered martensite matrix, imposed geometric constraints that suppressed localized plastic flow in the ferrite phase [23,24]. Collectively, these mechanisms promoted homogeneous strain distribution across the gauge length, delaying necking onset and enabling uniform elongation equivalent to the BM.

### 3.4. Impact Toughness and Service Reliability Assessment

**Figure 6:**
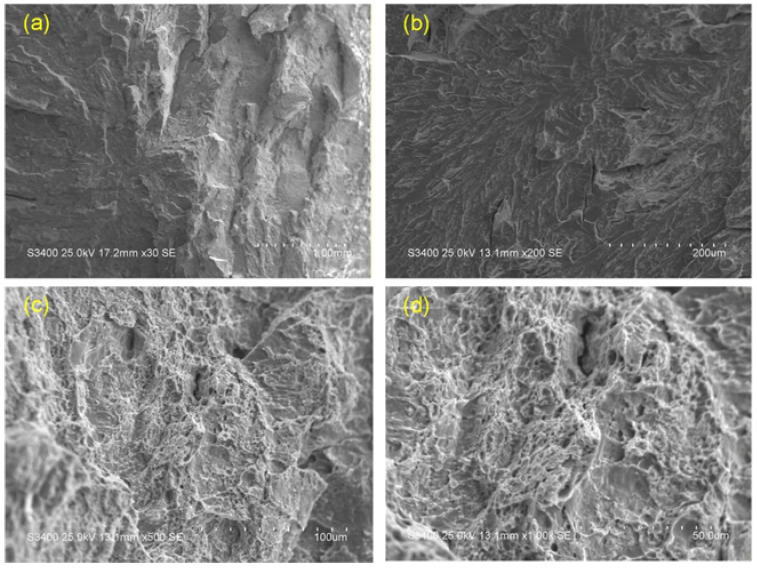
Impact fracture surface morphology of the WM region: (**a**,**b**) Low magnification; (**c**,**d**) High magnification.

**Figure 7:**
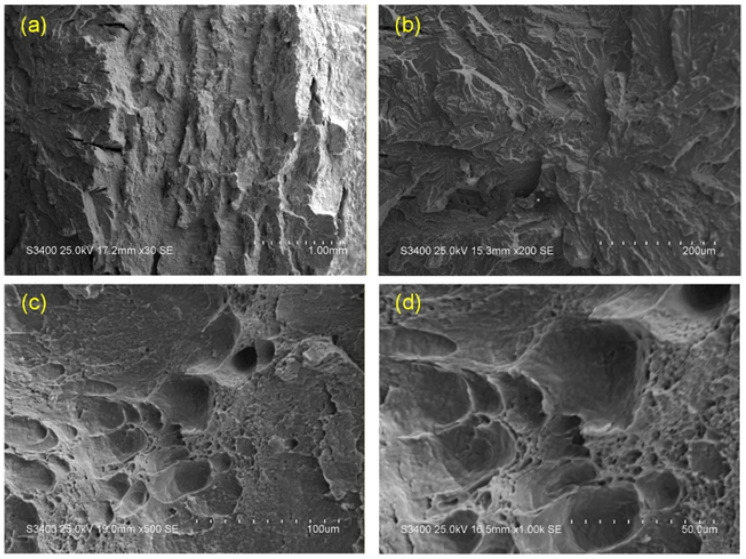
Impact fracture surface morphology of the HAZ: (**a**,**b**) Low magnification; (**c**,**d**) High magnification.

For ADS spallation target components, room-temperature Charpy V-notch impact energy serves as a critical indicator of baseline resistance to cleavage-initiated brittle fracture in the as-heat-treated condition. As summarized in Table 5 and corroborated by the fractographic analyses in Figure 6 and Figure 7, the WM exhibited an average absorbed energy of 171.2 ± 8.3 J. This toughness—substantially outperforming the base metal (71.3 J)—indicates an exceptional resistance to cleavage-initiated brittle fracture, while the HAZ averaged 74.4 ± 5.1 J. To elucidate the microscopic failure mechanisms governing these toughness differences, detailed SEM fractographic investigations were conducted. The low-magnification fractographs of the WM region (Figure 6a,b) reveal a highly tortuous and rugged macro-topography characterized by prominent tearing ridges, which indicates that the material underwent substantial plastic deformation and absorbed significant energy prior to final failure. Under higher magnification (Figure 6c,d), the fracture surface is predominantly occupied by dense clusters of well-developed, deep, and equiaxed ductile dimples. These dimple clusters, formed via microvoid coalescence around submicron second-phase particles, directly substantiate the outstanding room-temperature impact energy (171.2 J) and confirm a typical ductile fracture mode.

In contrast, the HAZ specimen displays a distinct mixed-mode (ductile-brittle) fracture lineage, as shown in Figure 7. The low-magnification views (Figure 7a,b) exhibit a relatively flat macro-topography featuring large, distinct cleavage facets and well-defined river patterns, which are indicative of localized cleavage-initiated brittle fracture within the coarsened or altered HAZ matrix. However, closer examination at higher magnification (Figure 7c,d) reveals that these flat cleavage zones are not completely continuous; instead, they are physically isolated and interconnected by narrow microscopic bands containing shallow shear dimples and plastic tearing ridges. This micro-topographical architecture explains why the HAZ impact energy (74.4 J) is lower than that of the WM, while demonstrating that the ductile matrix still retains sufficient capacity to interrupt continuous crack propagation. Figure 7 displays the representative fracture surface morphology of the HAZ specimen. Although this value is approximately 56% lower than that of the WM, it remains well above the ASME-specified toughness threshold (≥47 J), confirming sufficient fracture toughness margin to accommodate potential embrittlement mechanisms—including δ-ferrite coarsening or carbide precipitation—during extended high-temperature exposure.

This quantified toughness margin is directly relevant to predicting the component’s service life under thermal–mechanical cycling in ADS environments. In SIMP steel, retained δ-ferrite acts as a preferential nucleation site for the brittle Laves phase (Fe_2_W-type) during high-temperature exposure (>450 °C), as has been extensively confirmed by TEM-EDS analysis in long-term thermal aging studies of this alloy [25]. When δ-ferrite exhibits coarse, percolating morphology—characteristic of conventional high-heat-input welding—the precipitated Laves phase forms interconnected brittle networks along prior austenite grain boundaries, drastically reducing fracture toughness after thermal aging. In contrast, the narrow-gap automated TIG process constrained δ-ferrite to a volume fraction of 19.7 ± 1.2% with fine, discontinuous, and isolated particles dispersed uniformly throughout the matrix. This micro-topological architecture physically interrupts potential crack propagation paths, effectively functioning as a distributed microscopic crack-arrest mechanism. Such structural resilience is critical for ensuring component integrity in liquid lead–bismuth eutectic (LBE) environments, where stress corrosion cracking susceptibility is strongly correlated with localized brittle-phase continuity.

## 4. Conclusions

This study systematically investigates the microstructural evolution and mechanical performance of 7° narrow-gap automated TIG welded joints in 20 mm-thick SIMP steel forged plates—targeting structural integrity requirements for ADS spallation targets. The principal findings are as follows:

1. The narrow-gap automated TIG process enabled precise control of δ-ferrite morphology in the weld metal. Leveraging its intrinsic low thermal input and steep interfacial cooling gradient, the process suppressed macrosegregation of ferrite-stabilizing elements (Si, Cr) at the solid–liquid interface and kinetically inhibited δ-ferrite coarsening during non-equilibrium solidification. Quantitative analysis confirmed a retained δ-ferrite volume fraction of 19.7 ± 1.2%, with particles exhibiting fine, discontinuous, worm-like or island-shaped morphology uniformly dispersed within a tempered martensite matrix refined to ~22 μm prior austenite grain size—effectively eliminating coarse, continuous blocky δ-ferrite networks characteristic of conventional high-heat-input welding.
2. The joint exhibited no measurable local softening zones and achieved an exceptional balance between high-temperature strength retention and fracture toughness. Through-thickness microhardness profiling revealed uniform distribution (236 ± 5 HV) across the weld metal, BM, and heat-affected zone, indicating the absence of localized softening. This homogeneity arises from synergistic strengthening: (i) Hall–Petch strengthening via prior austenite grain and martensite lath refinement; and (ii) geometric constraint imposed by the dispersed δ-ferrite particles on plastic flow in the softer phase, thereby preventing strain localization. Consequently, the joint attained a room-temperature tensile strength of 820 MPa and retained 436 MPa (53.2% of ambient strength) at 550 °C—demonstrating excellent high-temperature structural stability—while sustaining tensile strength equivalent to the BM.
3. Room-temperature Charpy V-notch impact energy reached 171.2 ± 8.3 J in the weld metal, indicating outstanding impact toughness and structural defect tolerance. Critically, the fine, isolated δ-ferrite distribution physically interrupts potential crack propagation paths and suppresses percolation of the brittle Laves phase (Fe_2_W-type) during thermal aging (>450 °C). This microstructural design reduces susceptibility to stress corrosion cracking in liquid lead–bismuth eutectic (LBE) environments—where crack initiation is strongly correlated with brittle-phase continuity—thereby providing a robust metallurgical foundation for the full-life-cycle safety of SIMP steel thick-section components in ADSs.
4. Industrial Implications, Constraints, and Future Work: In an industrial manufacturing context, the automated NG-TIG process offers significant advantages over conventional arc welding techniques by enhancing production efficiency and ensuring exceptional microstructural homogeneity for thick-section SIMP steel components. The mechanical performance matches or exceeds current relevant literature values for martensitic heat-resistant steels. However, a primary constraint of the current methodology is that the structural integrity assessments were conducted under standard ex situ conditions. Future investigations will focus on evaluating the synergistic effects of high-intensity neutron irradiation and dynamic liquid lead–bismuth eutectic (LBE) corrosion on the microstructural stability—particularly the Laves phase evolution—to fully qualify these welded joints for long-term in-service ADS environments.

## Figures and Tables

**Table 1:** Chemical composition of 20Cr11W2VTaSi steel and welding wire (wt.%).

Element	C	Cr	Si	W	Mn	V	Ta
BM	0.14–0.25	10.0–12.0	1.0–2.0	1.0–2.0	≤1.0	0.15–0.30	0.1–0.2
Wire	0.14–0.2	10.0–12.0	0.8–1.0	1.9–2.2	1.2–1.4	0.25–0.30	0.15–0.18

**Table 2:** Room temperature mechanical properties of 20Cr11W2VTaSi steel.

Yield Strength (MPa)	Tensile Strength (MPa)	Elongation (%)	Impact Absorbed Energy (J)
629	849	20.4	71.3

**Table 3:** Process parameters for the automatic TIG welding.

Current (A)	Voltage (V)	Welding Speed (cm·min^−1^)
145~155	12~12.5	12~15

**Table 4:** Tensile properties of welded joints.

Test Temp	Rp0.2	Rm (MPa)	Elongation (%)	Fracture Location
Room Temp	616 ± 8	820 ± 9	23.7 ± 0.8	Weld
550 °C	368 ± 7	436 ± 9	-	Weld

**Table 5:** Room temperature impact toughness of welded joints.

Location	WM	HAZ
Impact Absorbed Energy (J)	171.2	74.4

## Data Availability

The original contributions presented in this study are included in the article. Further inquiries can be directed to the corresponding author.

## References

[1] Rubbia C., Rubio J.A., Carminati F., Fiétier N., Galvez J., Gelès C., Kadi Y., Klapisch R., Mandrillon P., Revo J.P. (1995). Conceptual Design of a Fast Neutron Operated High Power Energy Amplifier.

[2] Abderrahim H.A., Kupschus P., Malambu E., Benoit P., Van Tichelen K., Arien B., Vermeersch F., D’hondt P., Jongen Y., Ternier S. (2001). MYRRHA: A multipurpose accelerator driven system for research & development. Nucl.-Strum. Methods Phys. Res. Sect. A Accel. Spectrometers Detect. Assoc. Equip..

[3] Bowman C.D. (1998). Accelerator-driven systems for nuclear waste transmutation. Annu. Rev. Nucl. Part. Sci..

[4] Malerba L., Al Mazouzi A., Bertolus M., Cologna M., Efsing P., Jianu A., Kinnunen P., Nilsson K.-F., Rabung M., Tarantino M. (2022). Materials for sustainable nuclear energy: A european strategic research and innovation agenda for all reactor generations. Energies.

[5] Odette G.R. (2014). Recent progress in developing and qualifying nanostructured ferritic alloys for advanced fission and fusion applications. JOM.

[6] Wang J., Zhu K., Zhou J., Lu X. (2021). Effects of peak temperatures and cooling rates on delta ferrite formation and mechanical properties for heat affected zones in 9Cr-RAFM steel. Int. J. Press. Vessel. Pip..

[7] Setia P., Venkateswaran T., Tharian K.T., Jain J., Singh S.S., Shekhar S. (2022). Influence of Si content on the microstructure and mechanical properties of silicon stainless steel. Mater. Sci. Eng. A.

[8] Ma Z., Shen T., Zhu J., Jin P., Gao X., Wang Z. (2025). Microstructural evolution of SIMP steel under stress/lead–bismuth corrosion synergy. Mater. Des..

[9] Huang Q., Li C., Li Y., Chen M., Zhang M., Peng L., Zhu Z., Song Y., Gao S. (2007). Progress in development of China low activation martensitic steel for fusion application. J. Nucl. Mater..

[10] García-García V., Reyes-Calderón F., Frasco-García O.D., Alcantar-Modragón N. (2022). Mechanical behavior of austenitic stainless-steel welds with variable content of δ-ferrite in the heat-affected zone. Eng. Fail. Anal..

[11] Kumar R., Varma A., Kumar Y.R., Vashishtha H., Jain J., Neelakantan S. (2020). Optimization of post-weld heat treatment condition of arc-welded T91 steel tubes. Int. J. Press. Vessel. Pip..

[12] Xu K., Yin Y., Chen C. (2024). Research and application progress of welding technology under extreme conditions. Arch. Civ. Mech. Eng..

[13] Dell’Avvocato G., Bison P., Palmieri M., Ferrarini G., Palumbo D., Tricarico L., Galietti U. (2024). Non-destructive estimation of mechanical properties in Usibor^®^ 1500 via thermal diffusivity measurements: A thermographic procedure. NDT E Int..

[14] (2017). Standard Guide for Preparation of Metallographic Specimens.

[15] (2016). Standard Methods for Mechanical Testing of Welds.

[16] (2020). Standard Test Methods for Elevated Temperature Tension Tests of Metallic Materials.

[17] Zhang Y., He H., Wang H., Chen G., An X., Wang Y. (2021). Evolution of microstructure and mechanical properties of 9Cr ferrite/martensite steels with different Si content after long-term aging at 550 °C. J. Alloys Compd..

[18] Sirohi S., Pandey C., Goyal A. (2022). Brief study on δ-ferrite characterization: A review. Mater. Today Proc..

[19] Chen W.-B., Ding X.-B., Zhai L.-H., Zhou J.-M., Zhu J.-J., Zhu Q.-C., Jiang L., Li Z.-J., Dai Z.-M. (2024). Effect of δ-ferrite decomposition on the tensile properties of one modified 316H stainless steel: Experimental investigations and crystal plastic finite element simulations. Mater. Sci. Eng. A.

[20] Sam S., Das C., Ramasubbu V., Albert S., Bhaduri A., Jayakumar T., Kumar E.R. (2014). Delta ferrite in the weld metal of reduced activation ferritic martensitic steel. J. Nucl. Mater..

[21] Fedoseeva A., Tkachev E., Kaibyshev R. (2023). Advanced heat-resistant martensitic steels: Long-term creep deformation and fracture mechanisms. Mater. Sci. Eng. A.

[22] Ramakrishna T., Rao S.S., Naidu G.S. (2019). Strength and hardness of post-weld heat-treated thick section 7075 Al alloy friction stir welds. Mater. Test..

[23] Zhao Y., Liang M., Liu S., Zhang W. (2022). High-temperature fatigue behavior and cyclic deformation of a gradient nanostructured RAFM steel. Int. J. Fatigue.

[24] Falodun O., Oke S., Bodunrin M. (2025). A comprehensive review of residual stresses in carbon steel welding: Formation mechanisms, mitigation strategies, and advanced post-weld heat treatment techniques. Int. J. Adv. Manuf. Technol..

[25] Liu K., Wang D., Deng C., Gong B., Wu S. (2020). Improved microstructure heterogeneity and low-temperature fracture toughness of C–Mn weld metal through post weld heat treatment. Mater. Sci. Eng. A.

